# TNF-α regulates the proteolytic degradation of ST6Gal-1 and endothelial cell-cell junctions through upregulating expression of BACE1

**DOI:** 10.1038/srep40256

**Published:** 2017-01-16

**Authors:** Xiao Deng, Jun Zhang, Yan Liu, Linmu Chen, Chao Yu

**Affiliations:** 1 Institute of Life Science, Chongqing Medical University, Chongqing 400016, P. R. China

## Abstract

Endothelial dysfunction and monocyte adhesion to vascular endothelial cells are two critical steps in atherosclerosis development, and emerging evidence suggests that protein sialylation is involved in these processes. However, the mechanism underlying this phenomenon remains incompletely elucidated. In this study, we demonstrated that treatment with the proinflammatory cytokine TNF-α disrupted vascular endothelial cell-cell tight junctions and promoted monocyte endothelial cell adhesion. Western blotting and *Sambucus nigra* lectin (SNA) blotting analyses revealed that TNF-α treatment decreased α-2, 6-sialic acid transferase 1 (ST6Gal-I) levels and downregulated VE-Cadherin α-2, 6 sialylation. Further analysis demonstrated that TNF-α treatment upregulated β-site amyloid precursor protein enzyme 1 (BACE1) expression, thus resulting in sequential ST6Gal-I proteolytic degradation. Furthermore, our results revealed that PKC signaling cascades were involved in TNF-α-induced BACE1 upregulation. Together, these results indicated that the proinflammatory cytokine TNF-α impairs endothelial tight junctions and promotes monocyte-endothelial cell adhesion by upregulating BACE1 expression through activating PKC signaling and sequentially cleaving ST6Gal-I. Thus, inhibition of BACE1 expression may be a new approach for treating atherosclerosis.

Cardiovascular diseases, especially atherosclerosis, are the major cause of morbidity and mortality in patients with hypertension, obesity and diabetes^1^,^2^,^3^. Endothelial cell-to-cell junctions play a major role in the early stages of atherosclerosis, which are associated with inflammation and endothelial dysfunction^4^,^5^,^6^. The vascular endothelium is a layer of cells that lines the blood vessels and serves as the primary barrier between blood and tissues. Under chronic inflammatory conditions, endothelial impairment contributes to increased monocyte adhesion and the accumulation of extracellular matrix proteins, thus resulting in accumulation of atherogenic ApoB-containing lipoproteins at the arterial wall^7^,^8^,^9^. Therefore, studying the function of the human endothelium is helpful for investigating atherosclerosis development.

It is well known that protein glycosylation is an important post-translational modification. Studies have revealed that many glycoproteins participate in maintaining the normal endothelium and in the dynamic changes associated with endothelial pathophysiology^10^,^11^,^12^,^13^,^14^. Sialylation, a type of glycosylation characterized by the transfer of sialic acid to terminal galactose residues, is catalyzed by sialyltransferases^15^,^16^,^17^,^18^ and comprises the following two subtypes: β-galactoside α-2, 3-sialylation and β-galactoside α-2, 6-sialylation^19^. Recent studies have demonstrated that sialylation is necessary for adhesive molecule and chemokine receptor activity and is involved in the initiation and development of atherosclerotic lesions^13^. In a study by Döring Y *et al*., ST3Gal-IV-modified α-2, 3-sialylation has been found to decrease inflammatory leukocyte recruitment and to arrest during the early stages of atherosclerosis^20^. However, the roles of α-2, 6-sialylation in atherosclerosis development are poorly characterized.

Previous studies have devoted much attention to the biological functions of protein α-2, 6-sialylation, but the regulatory mechanisms controlling sialylation levels are poorly understood^21^,^22^. Eukaryotic cells need to maintain a sialylation steady state, because hypersialylation may result in cell dysfunction. Interestingly, β-site APP-cleaving enzyme 1 (BACE1) secretase has been widely identified as a protease responsible for 2, 6-sialic acid transferase 1 (ST6Gal-I)^21^,^23^,^24^ cleavage and secretion. BACE1 is highly expressed in the brain but is weakly expressed in endothelial cells^25^. Here, we investigated whether the BACE1 protein-degrading pathway is a novel mechanism that regulates ST6Gal-I and α-2, 6 sialylation levels in endothelial cells. To address this question, we investigated the roles of BACE1 in endothelial cells.

In the present study, we studied vascular endothelial cells to explore whether ST6Gal-I regulates cell adhesion junctions by adding sialic acids to VE-Cadherin in inflammatory environments. Furthermore, we evaluated whether BACE1 protein-degrading pathways can decrease protein α-2, 6-sialylation levels in vascular endothelial cells. To identify the mechanisms by which the proinflammatory factor TNF-α induces BACE1 upregulation in endothelial cells, we evaluated PKC/MEK/ERK pathway function after TNF-α treatment. We sought to address the question of how sialyltransferase influences the onset of atherosclerosis, because the answer may provide new insights regarding the prevention of vascular inflammation.

## Results

### The proinflammatory cytokine TNF-α disrupted the vascular epithelial barrier and promoted monocyte-endothelial functional adhesion

**Figure 1:**
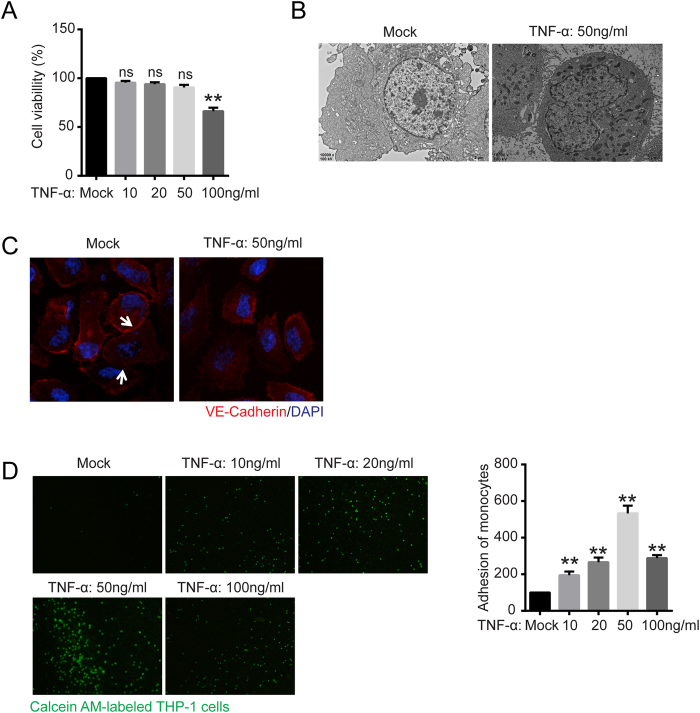
TNF-α impaired vascular epithelial cell tight junctions and increased vascular epithelial cell adhesion. (**A**) Cell viability was evaluated with CCK-8 assays. EA.hy926 cells were treated with 0, 10, 50, and 100 ng/ml TNF-α for 24 h and treated with WST-8 for 1 h at 37 °C. The absorbance at 450 nm was then measured with a microplate reader. ns, not significant versus untreated cells. (**B**) Transmission electron microscopy characterization of EA.hy926 cells with or without TNF-α treatment (50 ng/ml) for 24 h was performed (10000 × magnification). (**C**) Confocal immunofluorescence analysis of EA.hy926 cells treated with TNF-α using VE-Cadherin rabbit mAb (red) and DAPI (blue). (**D**) Monocyte adhesion to endothelial cells was quantified via monocyte adhesion assay. Calcein AM-labeled THP-1 cells were incubated with EA.hy926 cells in a 96-well plate for 1 h at 37 °C. The plate was then washed three times with PBS, and the fluorescence was measured. The data are the mean ± SD of three independent assays (**p < 0.01). Mock, EA.hy926 without treatment.

TNF-α, a proinflammatory cytokine induced in the early inflammatory response, promotes interactions between monocytes and vascular endothelial cells. To examine whether TNF-α has cytotoxic effects on human endothelial EA.hy926 cells, we used a CCK-8 assay to examine cell viability. CCK-8 is a convenient assay that utilizes the highly water-soluble tetrazolium salt WST-8 [2-(2-methoxy-4-nitrophenyl)-3-(4-nitrophenyl)-5-(2, 4-disulfophenyl)-2H-tetrazolium, monosodium salt], which produces a water-soluble formazan dye after reduction in the presence of an electron carrier. The results of the assay demonstrated that cell viability was not affected after TNF-α treatment up to a concentration of 50 ng/ml but was significantly decreased after 100 ng/ml TNF-α treatment (Fig. 1A). We next evaluated the effect of 50 ng/ml TNF-α on EA.hy926 cell tight junctions via transmission electron microscopy and confocal immunofluorescent analysis of the tight junction marker VE-Cadherin. As shown in Fig. 1B,C, the vascular epithelial barrier of EA.hy926 cells was disrupted after 50 ng/ml TNF-α treatment for 24 h. We next investigated the interactions between monocytes and vascular endothelial cells after TNF-α induction. As shown in Fig. 1D, significant increases in the degree of Calcein AM-labeled THP-1 adhesion to EA.hy926 occurred in the treated group compared with the mock-treated group after TNF-α exposure (10 to 50 ng/ml). However, the degree of Calcein AM-labeled THP-1 adhesion was slightly decreased when EA.hy926 cells were pretreated with 100 ng/ml TNF-α compared with 50 ng/ml TNF-α, probably because high concentrations of TNF-α result in EA.hy926 cell mortality.

### TNF-α treatment decreased ST6Gal-I levels and total protein α-2, 6 sialylation in EA.hy926 cells

**Figure 2:**
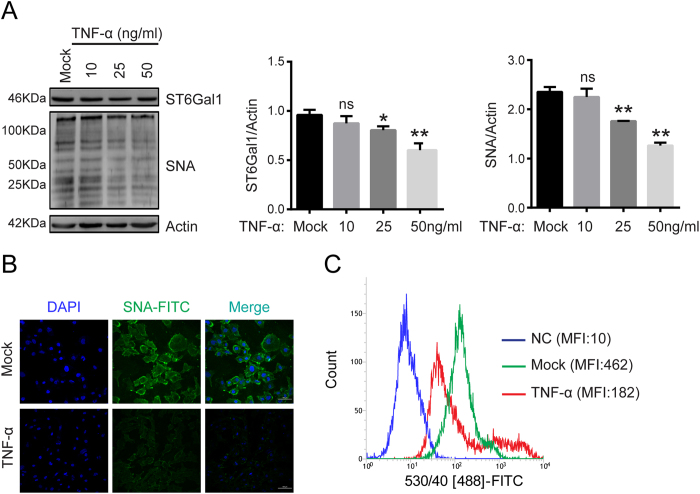
ST6Gal-I and total protein α-2, 6 sialylation levels were decreased in EA.hy926 cells after treatment with the proinflammatory cytokine TNF-α. (**A**) Western blotting and lectin blotting assays were conducted to characterize the levels of ST6Gal-I and total protein α-2, 6 sialylation in EA.hy926 cells treated with different concentrations of TNF-α. Actin was used as a loading control. The relative changes in the protein bands were measured, and the mock control was set as 100%, as shown. One typical result from three independent experiments is shown. ns, not significant, *p < 0.05 and **p < 0.01. After 0, 10, and 50 ng/ml TNF-α treatment for 24 h, immunoblotting was performed with anti-ST6Gal-I or SNA. An equal amount (30 μg) of total lysate from each sample was resolved by 10% SDS-PAGE, with β-actin serving as a control. The relative changes in the protein bands were measured, and the control was set to 100%, as shown in the data. One typical result from three independent experiments is shown. (**B**) EA.hy926 cells were treated with TNF-α (0, 50 ng/ml) or left untreated for 24 h. The cells then were incubated with FITC-labeled SNA and DAPI for 1 h. α-2, 6 sialylation expression was examined by confocal fluorescence microscopy (200 × magnification). Mock, EA.hy926 without treatment. (**C**) After TNF-α (0,50 ng/ml) treatment for 24 h, the cells were incubated with FITC-labeled SNA for 1 h. Flow cytometry assay was then used to determine α-2, 6 sialylation expression. The data are the mean ± SD of three independent assays (*p < 0.05, **p < 0.01). Mean fluorescence intensity (MFI) was quantified using BD FACS Software. NC, EA.hy926 cells unstained with FITC-labeled SNA. Mock, EA.hy926 without treatment.

Sialic acid plays an important role in endothelial dysfunction. To assess whether TNF-α treatment altered total protein sialylation levels in EA.hy926 cells, we used flow cytometry to detect total protein α-2, 6 or α-2, 3 sialylation levels, by using FITC-labeled SNA or FITC-labeled MAA, lectins that recognize α-2,6 and α-2,3 sialic acid, respectively. Total protein α-2, 3 sialylation levels were not significantly altered after TNF-α treatment (Supplementary Fig. 1). However, total protein α-2, 6 sialylation levels were notably decreased after TNF-α treatment (Fig. 2C). Total protein α-2, 6 sialylation levels were also detected by lectin staining. The results indicated that SNA staining intensity was visually weaker in TNF-α-treated EA.hy926 cells than in mock-treated cells (Fig. 2B). α-2, 6 sialylation was regulated by ST6Gal-I levels. We evaluated the effect of TNF-α on ST6Gal-I protein levels. As indicated in Fig. 2A, TNF-α clearly decreased the ST6Gal-I levels. Together, these results demonstrate that TNF-α decreased ST6Gal-I and total protein α-2, 6 sialylation levels in EA.hy926 cells.

### VE-Cadherin α-2, 6 sialylation was decreased after TNF-α treatment

**Figure 3:**
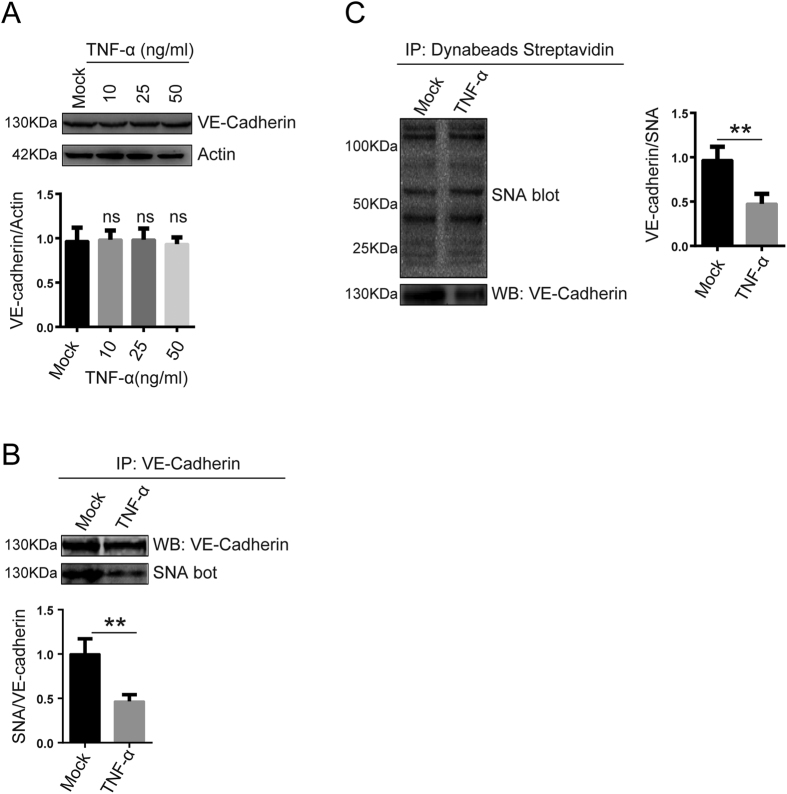
VE-Cadherin α-2, 6 sialylation levels were decreased under proinflammatory conditions. (**A**) VE-cadherin levels in EA.hy926 cells treated with TNF-α (0, 10, 20, and 50 ng/ml) for 24 h were examined via western blot assays. (**B**) VE-Cadherin α-2, 6 sialylation levels in EA.hy926 cells were decreased after TNF-α (50 ng/ml) treatment for 24 h. VE-cadherin was immunoprecipitated with anti-human VE-cadherin polyclonal antibodies. VE-cadherin immunoprecipitates were analyzed via SNA blotting using biotinylated SNA and HRP-labeled Streptavidin. (**C**) Proteins modified by α-2, 6 sialylation in EA.hy926 cells were recognized via biotinylated SNA blotting and then precipitated with Dynabeads Streptavidin. VE-Cadherin levels in the precipitates were analyzed by western blotting using antibodies against VE-cadherin. The data are the mean ± SD of three independent assays (**p < 0.01).

VE-cadherin is the major endothelial tight junction marker; it mediates adhesion between monocytes and endothelial cells and may mediate vascular intracellular signaling pathways. We hypothesized that VE-Cadherin might be involved in TNF-α-induced endothelial dysfunction. As shown in Fig. 3A, VE-cadherin protein levels were not affected after TNF-α treatment for 24 h, thus indicating that endothelial dysfunction was not caused by VE-cadherin expression (Fig. 3A). It has been reported that VE-Cadherin can be sialylated. Therefore, we evaluated whether TNF-α affects VE-Cadherin sialylation. The results demonstrated that VE-Cadherin α-2, 6 sialylation was decreased after TNF-α treatment for 24 h in treated cells compared with untreated control cells (Fig. 3B,C).

### TNF-α treatment upregulated BACE1 expression, thus resulting in ST6Gal-I proteolytic degradation

**Figure 4:**
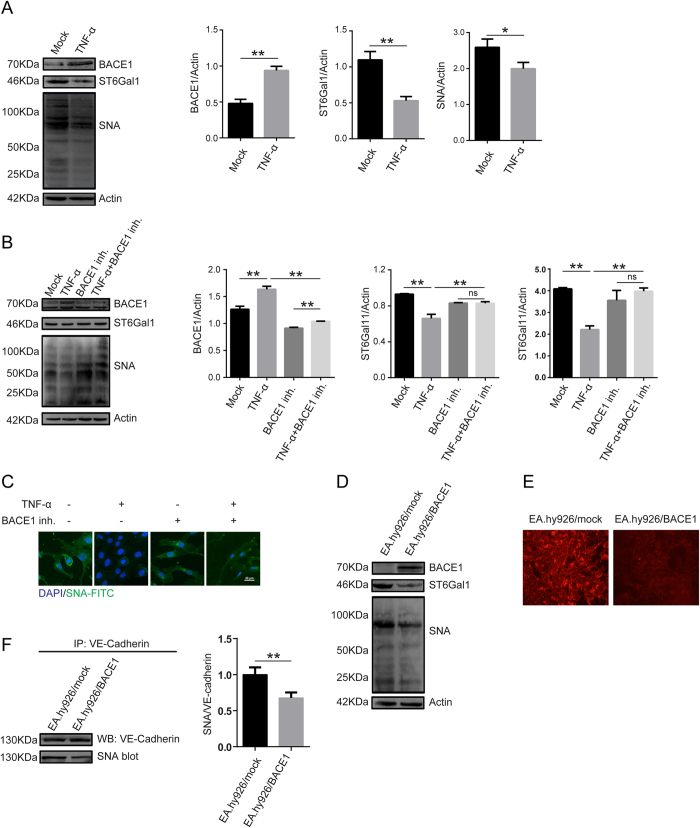
TNF-α regulates ST6Gal-I levels through BACE1. (**A**) EA.hy926 cells were treated with TNF-α for 24 h, and then BACE1, ST6Gal-I and total protein α-2, 6 sialylation levels were detected by western blotting and SNA blotting. Actin was used as a loading control. The data are the mean ± SD of three independent assays (**p < 0.01, *p < 0.05). (**B**) The cells were pretreated with 1 μM BACE1 inh. for 24 h and then stimulated with TNF-α (50 ng/ml) for 24 h and analyzed by western blotting and SNA blotting. The data are the mean ± SD of three independent assays (**p < 0.01). ns, not significant. (**C**) The cells were pretreated with 1 μM BACE1 inh. for 24 h and then stimulated with TNF-α (50 ng/ml) for 24 h. The cells were incubated with FITC-labeled SNA and DAPI for 1 h. Proteins α-2, 6 sialylation levels were examined under confocal fluorescence microscopy (600 × magnification). (**D**) EA.hy926 cells were inoculated with a BACE1-overexpressing lentivirus and selected with puromycin for 4 weeks. BACE1, ST6Gal-I and total protein α-2, 6 sialylation levels were then detected by western blotting and SNA blotting. EA.hy926/Mock and EA.hy926 cells transduced with control lentivirus. EA.hy926/BACE1 and EA.hy926 cells transduced with BACE1-overexpressed lentivirus. (**E**) Total protein α-2, 6 sialylation levels in cells overexpressing BACE1 were examined by SNA staining. Because they were transduced with lentiviruses carrying an EGFP marker gene, the EA.hy926 cells were sequentially incubated with biotinylated SNA and Avidin-RBITC, which specifically binds biotinylated SNA. RBITC fluorescence signals were detected under confocal fluorescence microscopy. (**F**) Sialylated VE-cadherin levels were decreased after BACE1 overexpression. Cell lysates were immunoprecipitated with anti-VE-cadherin antibody and analyzed by SNA blotting. The data are the mean ± SD of three independent assays (**p < 0.01).

BACE1 has been identified as a protease responsible for the cleavage of Golgi-resident ST6Gal-I. To confirm the relevance of BACE1 in ST6Gal-I proteolytic degradation in EA.hy926 cells, we conducted western blotting and SNA blotting. As shown in Fig. 4A, TNF-α treatment significantly increased BACE1 protein expression and decreased ST6Gal-I and α-2, 6 sialylation levels in the treated group compared with the mock-treated group. More importantly, BACE1 inhibitor pretreatment markedly inhibited TNF-α-induced decreases in ST6Gal-I and total protein α-2, 6 sialylation levels in EA.hy926 cells (Fig. 4B,C).

To investigate the ST6Gal-I proteolytic degradation-mediated effects of BACE1 on α-2, 6 sialylation further, we constructed a BACE1-overexpressing lentivirus and transduced the lentivirus into EA.hy926 cells (known as EA.hy926/BACE1 cells). Higher BACE1 expression levels were detected in EA.hy926/BACE1 cells than in other cells, but ST6Gal-I and total protein α-2, 6 sialylation levels were decreased in EA.hy926/BACE1 cells compared with other cells (Fig. 4D,E). Simultaneously, VE-Cadherin sialylation levels were dramatically decreased in EA.hy926/BACE1 cells compared with other cells (Fig. 4F). These results indicated that BACE1 was associated with TNF-α-induced ST6Gal-I proteolytic degradation and that α-2, 6 sialylation of VE-Cadherin decreased in EA.hy926 cells.

Finally, further experiments in primary HUVECs confirmed the regulatory effects of BACE1 on ST6Gal-I and α-2, 6 sialylation downregulation under inflammatory conditions (Supplementary Fig. 2).

### BACE1 is functionally important in regulating vascular endothelial cell functions

**Figure 5:**
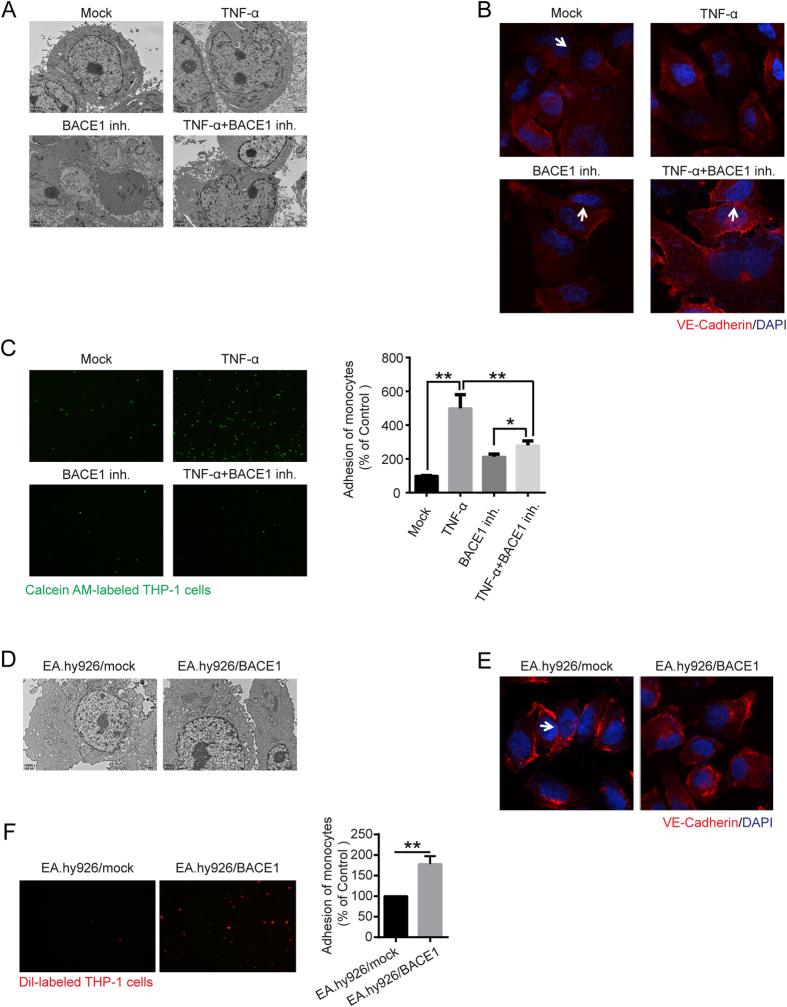
Tight junctions and cell adhesion were affected by BACE1. (**A**) BACE1 inh. (1 μM) was administered as pretreatment for 24 h, and then the cells were stimulated with TNF-α (50 ng/mL) for 24 h. EA.hy926 cell tight junctions were detected by transmission electron microscopy (10000 × magnification). (**B**) EA.hy926 cell tight junctions were also detected by confocal immunofluorescence analysis using anti-VE-cadherin antibody (Red). Blue, DAPI. (**C**) After BACE1 inh. (1 μM) treatment, cell adhesion assay was performed in EA.hy926 cells (100 × magnification). The data are the mean ± SD of three independent assays (**p < 0.01). (**D**) Transmission electron microscopy analysis was used to identify BACE1-overexpressing EA.hy926 cell tight junctions (10000 × magnification). (**E**) Immunofluorescent analysis of the tight junction protein VE-cadherin. Red, VE-cadherin. Blue, DAPI. (**F**) Monocyte adhesion to endothelial cells was quantified by monocyte adhesion assay. Dil-labeled THP-1 cells were incubated with endothelial cells in 96-well plates for 1 h at 37 °C. The plates were then washed three times with PBS, and the fluorescence was measured. The data are the mean ± SD of three independent assays (**p < 0.01).

We explored whether BACE1 mediates vascular endothelial cell functions. As indicated in Fig. 5A,B, BACE1 inhibitor pretreatment clearly disrupted the EA.hy926 cell vascular epithelial barrier. BACE1 inhibition markedly inhibited TNFα-induced endothelial dysfunction and monocyte adhesion (Fig. 5C). However, BACE1 overexpression in EA.hy926/BACE1 cells induced considerable increases in endothelial cell tight junction disruption and monocyte adhesion compared with those in EA.hy926 cells, thus demonstrating that BACE1 expression is correlated with regulation of vascular endothelial cell functions.

### Activation of the PKC/MEK/ERK signaling cascades is associated with BACE1 expression under proinflammatory conditions

**Figure 6:**
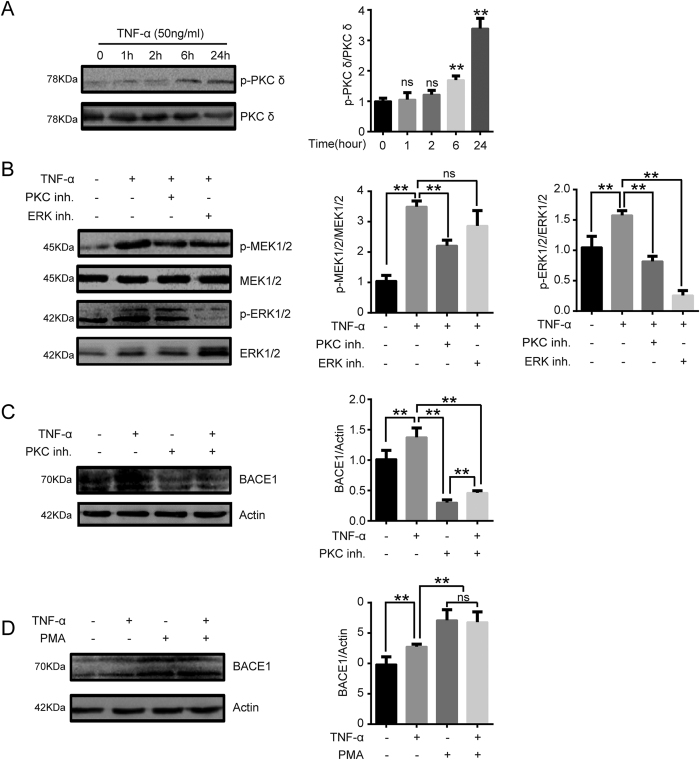
BACE1 expression induced by the proinflammatory cytokine TNF-α is PKC-dependent. (**A**) EA.hy926 cells were treated with TNF-α (50 ng/ml) for the indicated time periods. Western blotting was conducted to characterize phospho-PKC δ levels. Quantification of normalized densities for p-PKC δ and PKC δ is shown. The graphs represent the relative activity of these kinases for three independent experiments. ns, ns, not significant versus untreated cells. **p < 0.01 versus untreated cells. (**B**) EA.hy926 cells were stimulated with TNF-α (50 ng/ml) with or without PKC inhibitor (10 μM) or ERK inhibitor (20 μM) for 24 h, and MEK1/2, phospho-MEK1/2, ERK1/2 and phospho-ERK1/2 levels were analyzed via western blotting assays. ns, not significant. **p < 0.01. (**C**,**D**) Effect of the PKC pathway on BACE1 expression in EA.hy926 cells. The cells were pretreated with a PKC activator (10 μM) for 6 h or PKC inhibitor (10 μM) for 30 min and then incubated with TNF-α (50 ng/ml) for 24 h. BACE1 expression levels in EA.hy926 cells were analyzed via western blotting. ns, not significant. **p < 0.01.

A recent study has demonstrated the existence of a positive correlation between PKC signaling cascade activation and increased BACE1 expression in fibroblasts and neuroblastoma cells, thus suggesting that PKC signaling cascades may be involved in BACE1 expression in vascular endothelial cells^26^,^27^. To gain insight into the potential signaling pathways regulating BACE1 expression and protein sialylation levels, phospho-PKC δ and PKC δ protein levels were determined via western blotting analysis. The results of this analysis showed that PKC δ phosphorylation levels in EA.hy926 cells increased in a time-dependent manner after TNF-α treatment (Fig. 6A) and that TNF-α treatment also increased MEK1/2 and ERK1/2 phosphorylation levels. However, PKC inhibitor pretreatment inhibited MEK1/2 and ERK1/2 activation, thus indicating that TNF-α may induce Raf-MEK/ERK signaling pathway activation in vascular endothelial cells via PKC-dependent pathways (Fig. 6B). We determined the effects of PKC signaling cascades on BACE1 expression in EA.hy926 cells. As shown in Fig. 6C,D, PKC inhibition significantly suppressed BACE1 expression, whereas treatment with PMA, a PKC activator, promoted BACE1 expression. These data indicate that the PKC/MEK/ERK pathway may be involved in regulating BACE1 expression under proinflammatory conditions.

## Discussion

In this study, we intensively studied the possible effects of sialylation modification on endothelial dysfunction and monocyte-endothelial cell adhesion. We also demonstrated the mechanism by which sialylation is regulated in TNFα-treated vascular endothelial cells. We found that BACE1 upregulation in TNFα-treated endothelial cells leads to ST6Gal-I proteolysis and results in endothelial dysfunction and monocyte-endothelial cell adhesion.

The endothelium, which forms the inner cellular lining of blood vessels and lymphatics, is a highly metabolically active organ that is involved in atherosclerosis development and controls vasomotor tone, barrier function, monocyte adhesion, and trafficking and inflammation^28^,^29^. Our study demonstrated that the proinflammatory cytokine TNF-α impaired endothelial cell-to-cell tight junctions and promoted monocyte adhesion to endothelial cells (Fig. 1). ECs have three surfaces as follows: cohesive, adhesive, and luminal. The cohesive surface joins ECs to each other, facilitates transport processes; it consists of the following specialized intercellular junctions: gap junctions, tight junctions, and adherens junctions and syndesmos junctions^30^,^31^. The adhesive surface of ECs adheres to the basal lamina, and the luminal surface of the vascular endothelium consists of molecules and specific binding proteins that regulate the trafficking of circulating blood cells^32^. Therefore, endothelial dysfunction is associated with endothelial cell-to-cell junction disruption.

Although previous studies have focused on endothelial cell surface-bound chemokine expression in endothelial dysfunction and atherosclerosis development, cell adhesion molecules and chemokine receptors are also glycoproteins, thus suggesting that other regulatory mechanisms, such as post-translational glycosylation, may contribute to their activities^33^. Aberrant sialylation has been correlated with leukocyte arrest during inflammation^34^,^35^. Our results revealed that total protein α-2, 6 sialylation was decreased in response to TNF-α-induced inflammation (Fig. 2), which in turn correlated with endothelial tight junction disruption and monocyte-endothelial cell adhesion. Whether α-2, 6 sialylation regulates interactions between monocytes and endothelial cells remains unknown. Endothelial tight junctions have been studied extensively^5^,^36^,^37^. VE-Cadherin, an important target protein, contributes to the maintenance of vascular integrity and promotes endothelial cell-to-cell adhesion^28^. Our data showed that VE-Cadherin expression levels were not significantly changed, but VE-Cadherin 2, 6 sialylation levels were markedly decreased after TNF-α treatment (Fig. 3), thus indicating that VE-Cadherin 2, 6 sialylation may be involved in the interactions between monocytes and endothelial cells.

BACE1 is a crucial protease in the pathogenesis of Alzheimer’s disease. BACE1 is highly expressed in the brain; however, its function outside of the brain has not been elucidated^23^. Previous studies have reported that cerebral atherosclerosis and arteriolosclerosis are highly associated with Alzheimer’s disease and that BACE1 is upregulated in congestive heart failure^38^,^39^. Furthermore, many previous reports have indicated that BACE1 plays an important role in ST6Gal-I cleavage. To address the hypothesis that BACE1 is upregulated in vascular endothelial cells under inflammation, decreases ST6Gal-1 and protein α2, 6 sialylation levels, and finally promotes monocyte-endothelial cell adhesion, we investigated the potential role of BACE1 in endothelial cells. The results showed that BACE1 protein levels were dramatically upregulated after TNF-α treatment, thereby resulting in ST6Gal-I cleavage and dramatically decreasing α-2, 6 sialylation in vascular endothelial cells (Fig. 4A and Supplementary Fig. 2). In addition, the hypothesis that BACE-1 is one of the proteases responsible for ST6Gal-1 cleavage and α-2, 6 sialylation was proven by experiments using β-secretase inhibitors or overexpressing BACE1 (Fig. 4B–F). Moreover, altered BACE1 levels led to changes in endothelial tight junction structure and monocyte-endothelial cell adhesion (Fig. 5). The results of these experiments indicated that TNF-α upregulated the expression of BACE1, which was in turn associated with changes in endothelial cell-cell junctions mediated by ST6Gal-I cleavage.

In subsequent experiments, we also investigated the molecular mechanism by which TNF-α induces BACE1 upregulation. Many studies have confirmed that PKC signaling is involved in BACE1 upregulation in neuroblastoma and fibroblast cell lines^26^,^27^. It is well known that PKC activation is induced by TNFR members, such as TNFR1, FAS (TNFRSF6), OX40, and TRAIL-R1/2^40^. However, whether PKC signaling activation induced by TNF-α has any direct regulatory effects on BACE1 in endothelial cells remains unclear. Our study showed that PKC/MEK/ERK signaling cascade activation by TNF-α is associated with BACE1 upregulation (Fig. 6). This finding provides further strong support for the hypothesis that PKC signaling is an important upstream effector in TNF-α -induced cell pathways that lead to increased BACE1 expression.

In conclusion, we showed that the proinflammatory cytokine TNF-α impairs endothelial tight junctions and promotes monocyte adhesion to endothelial cells by upregulating BACE1 expression via the activation of PKC signaling and sequential cleavage of ST6Gal-I. This study provides novel insights into the BACE1 protein-degrading pathways in endothelial cells and suggests that inhibiting BACE1 expression may represent a new approach for treating atherosclerosis.

## Materials and Methods

### Reagents and chemicals

Dulbecco’s modified Eagle’s medium (DMEM) and fetal bovine serum (FBS) were purchased from Invitrogen (Carlsbad, CA, USA). A Cell Counting Kit-8 (CCK8) was purchased from Beyotime (Shanghai, China), β-secretase inhibitor IV (BACE1 inh.) was purchased from Biochemicals (Solon, OH, USA), and phorbol-12-myristate-13-acetate (PMA) was purchased from Sigma (St. Louis, MO, USA). Polyclonal antibodies against phospho-MEK, phospho-PKCδ, phospho-ERK, BACE1, MEK, PKCδ, and ERK and the monoclonal antibody against β-actin were purchased from Cell Signaling Technology (Danvers, MA, USA), and the anti-ST6GAL1 antibody was purchased from Santa (Dallas, Texas, USA).

### Cell culture

The EA.hy926 cell line was purchased from the Type Culture Collection of the Chinese Academy of Sciences, Shanghai, China (No. GNHu39). EA.hy926 cells were established by fusing primary human umbilical vein endothelial cells (HUVECs) with a thioguanine-resistant A549 clone via polyethylene glycol (PEG) exposure. The EA.hy926 cells were cultured in DMEM supplemented with 10% fetal bovine serum in 5% CO_2_ at 37 °C in a humidified incubator. Primary HUVECs were a gift from Dr. Jianghong Yan (Institute of Life Science, Chongqing Medical University) and were cultured in endothelial cell medium (Sciencell, California, USA) supplemented with 10% fetal bovine serum in 5% CO2 at 37 °C.

### Cell viability assay

Cell viability was analyzed via CCK8 (Sigma-Aldrich, 96992) assays. Briefly, the EA.hy926 cells were plated in a 96-well plate at a density of 10,000 cells per well and cultured overnight. The cells were then treated with gradient dilutions of TNF-α (0, 10, 20, 50, 100 ng/ml) or 50 ng/ml TNF-α (0, 6 h, 12 h, 24 h, 48 h) in 100 μl of medium. One hundred microliters of CCK-8 solution was added to each well of the plate after 24 h, and the plate was incubated at 37 °C for 4 h. The absorbance of each well was measured at 450 nm using a VERS Amax Microplate Reader (Molecular Devices Corp, Sunnyvale, CA).

### Immunofluorescence

The EA.hy926 cells were seeded onto coverslips (Fisher Scientific) in 24-well plates (Corning) containing DMEM. After being treated with TNF-α for 24 h, the cells were fixed with 4% paraformaldehyde and blocked with 10% normal goat serum in PBS at room temperature for 1 h. Then, the cells were incubated with rabbit monoclonal anti-VE-cadherin antibodies (CST) overnight at 4 °C. After incubation, the cells were washed three times with PBS and incubated with AlexaFluor 594-conjugated goat anti-rabbit IgG and 10 μM DAPI (Molecular Probes) and were then washed three times in PBS. Finally, the cells were visualized with a confocal microscope.

For SNA staining, the cells were fixed with 4% paraformaldehyde for 10 min and blocked with 10% BSA for 1 h at room temperature (RT). The cells were then incubated with FITC-labeled SNA or FITC-labeled MAA (Vector Laboratories, USA) at 37 °C for 1 h. The fluorescence pattern was analyzed by confocal fluorescence microscopy.

### Cell adhesion assay

Briefly, the EA.hy926 cells were seeded in 96-well plates at 1 × 10^5^ cells/ml overnight and stimulated with the indicated concentration of TNF-α for 24 h in the presence of BACE1 inh. For fluorescence labeling, THP-1 cells were incubated with 10 μl of Calcein AM (Beyotime, Shanghai, China) at 37 °C for 1 h and then harvested by centrifugation (1,000 × g, 5 min) and washed three times with PBS. The Calcein AM-labeled THP-1 cells were then suspended in medium and added to the EA.hy926 cells after being treated with TNF-α at the indicated concentrations. After 1 h, the non-adhering THP-1 cells were washed twice with PBS, and the THP-1 cells that were adherent to the EA.hy926 cells were observed and counted under a NIKON-TE2000-U microscope.

### Western blot assay

Protein concentrations were measured with a BCA assay kit (Beyotime, Shanghai China). Equal amounts of denatured protein were subjected to 10% SDS-PAGE and blotted onto PVDF membranes (Millipore, Bedford, MA, USA). Antibodies against ST6Gal-I (Santa Cruz Biotech, USA), BACE1, VE-Cadherin, phospho-PKCδ, PKCδ, phospho-MEK1/2, MEK1/2, phospho-ERK44/42, ERK44/42 (Cell Signaling Technology, USA) and β-actin (Beyotime, Shanghai, China) were used as primary antibodies, and horseradish peroxidase-conjugated goat α-rabbit IgG (Beyotime, Shanghai, China) was used as the secondary antibody. Detection was performed using an ECL kit (Millipore, USA), according to manufacturer’s instructions. The relative amount of protein was determined by densitometry using Labworks software.

### Lectin blot analysis

Cell lysates containing equal amounts of denatured proteins were subjected to 10% SDS-PAGE and transferred to PVDF membranes. Identification was performed with 2 μg/ml biotinylated Sambucus nigra lectin (SNA) (Vector Laboratories, USA), which specifically recognizes the attachment of sialic acid to terminal galactose via α-2, 6-linkages. HRP-labeled Streptavidin (Bioss, Beijing, China) was used in biotinylated-SNA recognition. Finally, the blots were detected using an ECL detection system.

### Immunoprecipitation (IP) assays

Cells in a T25 cell culture flask (NEST) were lysed with 1 ml of IP lysis buffer (25 mM Tris, 150 mM NaCl, [pH 7.2]) containing 10 μl of phenylmethylsulfonyl fluoride (PMSF) for 30 min at 4 °C. After the lysates were centrifuged at 12,000 × g for 30 min at 4 °C, the supernatant was collected, added into 2 μl of anti-VE-cadherin antibody (Cell Signaling Technology, USA) and incubated at 4 °C overnight. Thereafter, protein A+G agarose beads (Beyotime) were added to the mixture and incubated at 4 °C for 6 h. The beads were then centrifuged at 3,000 × g for 5 min at 4 °C for collection and washed six times with IP lysis buffer. After the addition of 10 μl of 5 × SDS loading buffer, the beads were boiled in water for 10 min and centrifuged at 13,000 × g for 10 min at RT. The precipitated proteins were subjected to western blotting or SNA blotting assays. To precipitate sialyted proteins in EA.hy926 cells, the cell lysates were collected and added to biotinylated SNA. After incubation overnight at 4 °C, the biotinylated SNA-binding proteins were precipitated with Dynabeads Streptavidin (Thermo Fisher Scientific). VE-Cadherin levels in the precipitates were analyzed by western blotting using antibodies against VE-Cadherin.

### Electron microscopy assays

For electron microscopy assay, cells on a Transwell insert membrane were fixed in 2.5% glutaraldehyde and 1% tannic acid in 0.1 M sodium cacodylate buffer (pH 7.4) for 1 h at RT, washed in 0.1 M cacodylate buffer, and then postfixed with 1% OsO_4_ in the same buffer. The samples were stained en bloc with 1% uranyl acetate, dehydrated in ethanol, and embedded in Epon 812 (Electron Microscopy Sciences, USA). The sections were stained with lead citrate and examined with a JEOL 1200 EX electron microscope.

### BACE1 overexpression

BACE1-overexpressing lentiviruses were obtained from Genepharmagps (Shanghai, China). EA.hy926 cells were inoculated with 1 μl of virus in 60-mm dishes using PloyFect Transfection Reagent (Qiagen, Valencia, CA), according to the manufacturer’s instructions. After 4 weeks of selection, the cell line stably expressing BACE1 was established.

### Flow cytometry

Cells were grown on 60-mm dishes for 24 h before being detached with 5 mM EDTA in PBS and resuspended as single-cell suspensions in fluorescence-activated cell sorter buffer (PBS containing 1% bovine serum albumin and 0.05% sodium azide) at a density of 1 × 10^7^ cells/ml. The cells were incubated with SNA-FITC (2 μg/ml) or MAA-FITC (2 μg/ml) for 1 h at RT, washed three times, and suspended in fluorescence-activated cell sorting buffer. Flow cytometry data were acquired using a BD influx Cell Sorter (BD Biosciences, USA).

### Statistical analysis

Data are presented as the mean ± S.E.M. Comparisons between treatments were performed using GraphPad PRISM software, version 5.00 (San Diego, CA), using one-way ANOVA with Tukey’s post hoc test or two-way ANOVA with Bonferroni’s post hoc test. Statistical significance was accepted when p < 0.05.

## Additional Information

**How to cite this article:** Deng, X. *et al*. TNF-α regulates the proteolytic degradation of ST6Gal-1 and endothelial cell-cell junctions through upregulating expression of BACE1. *Sci. Rep.* **7**, 40256; doi: 10.1038/srep40256 (2017).

**Publisher's note:** Springer Nature remains neutral with regard to jurisdictional claims in published maps and institutional affiliations.

## Supplementary Material

Supplementary Information
