# Supplementary material for: TNF-α regulates the proteolytic degradation of ST6Gal-1 and endothelial cell-cell junctions through upregulating expression of BACE1
Source: Sci Rep. 2017 Jan 16;7:40256. doi: 10.1038/srep40256 (PMC5238365; doi:10.1038/srep40256)

TNF-α regulates the proteolytic degradation of ST6Gal-1 and endothelial cell-cell junctions through upregulating expression of BACE1

Xiao Deng[1], Jun Zhang[1], Yan Liu[1], Linmu Chen[1], Chao Yu[1]*

[1]Institute of Life Science, Chongqing Medical University, Chongqing 400016, P. R. China

X.D. and J.Z. contributed equally to this study

*Corresponding author: Prof. Chao Yu,

Email: yuchaom@163.com

Phone: (86) 23-68485589

Fax: (86) 23-68486294

Full address: Box #174, Institute of Life Sciences, Chongqing Medical University, No. 1 Yixueyuan Road, Yuzhong District, Chongqing 400016, P. R. China.

Supplementary Figure 1

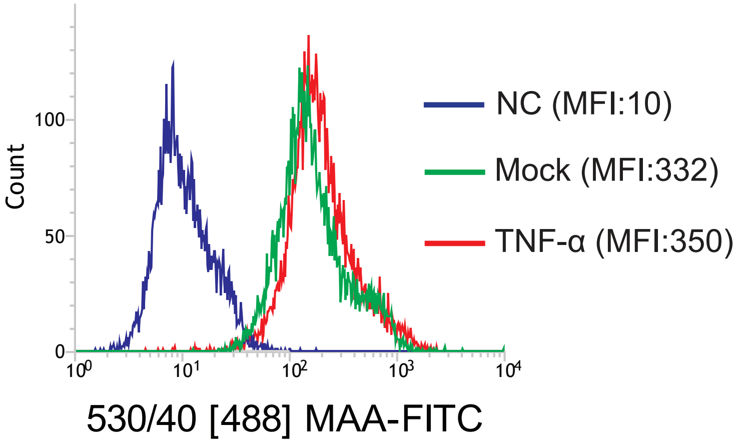

Supplementary Fig. 1 Flow cytometry data for total protein α-2, 3 sialylation levels in EA.hy926 cells treated with TNF-α. EA.hy926 cells were treated with TNF-α (50 ng/ml) for 24 h and then incubated with MAA-FITC (2 μg/ml) for 1 h at room temperature. After being washed three times in PBS, the cells were analyzed using a BD Influx Cell Sorter (BD Biosciences, USA). The graphs represent the relative results of three independent assays. MFI, mean fluorescence intensity. NC, EA.hy926 cells unstained with FITC-labeled SNA. Mock, EA.hy926 without treatment.

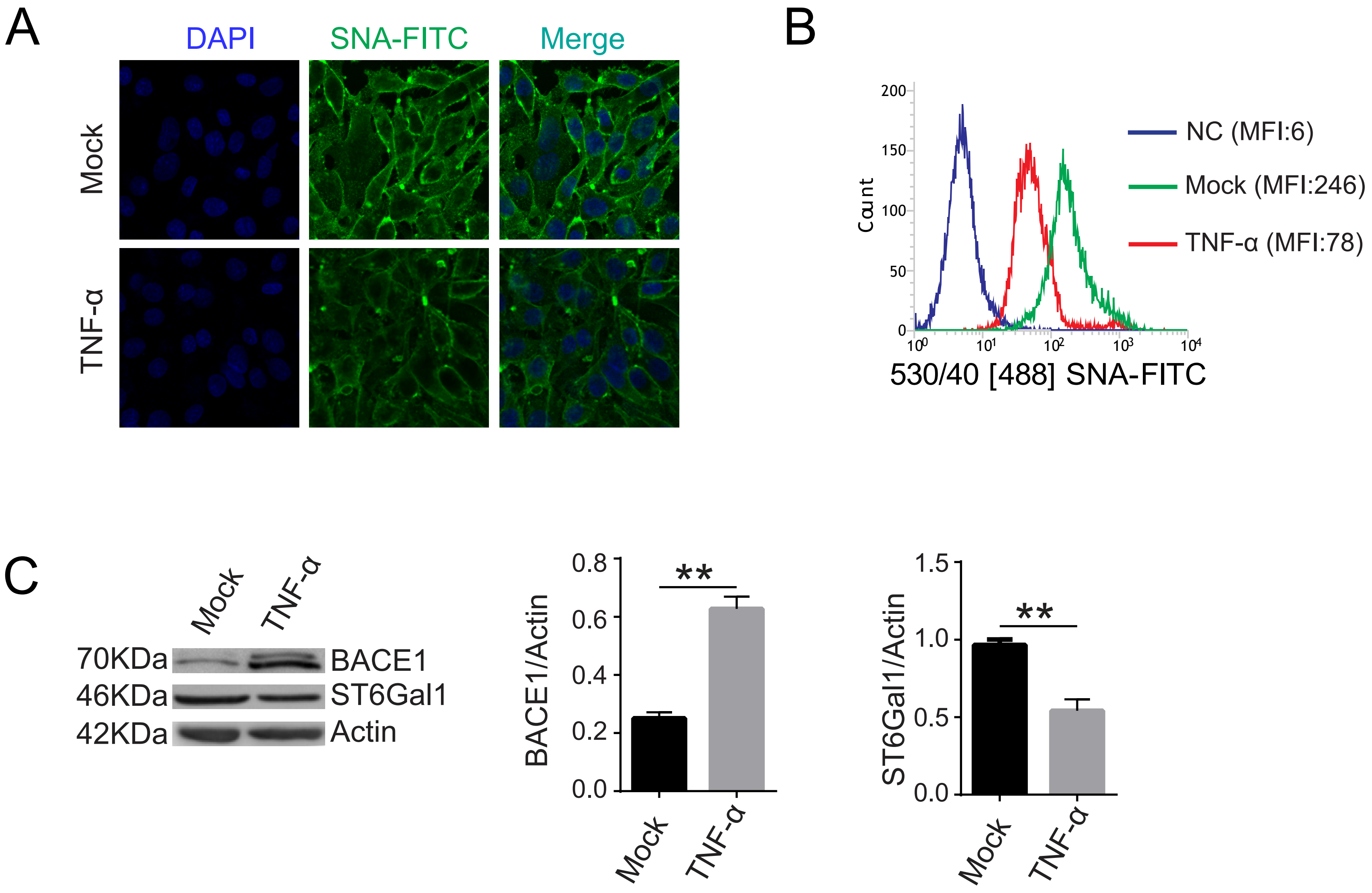
A
DAPI
SNA-FITC
Merge
Mock
TNF-α
B
200
150
100
50
0
Count
10^0
10^1
10^2
10^3
10^4
530/40 [488] SNA-FITC
NC (MFI:6)
Mock (MFI:246)
TNF-α (MFI:78)
C
Mock
TNF-α
70KDa
46KDa
42KDa
BACE1
ST6Gal1
Actin
BACE1/Actin
0.8
0.6
0.4
0.2
0.0
**
ST6Gal1/Actin
1.5
1.0
0.5
0.0
**

Supplementary Fig. 2 TNF-α treatment decreases ST6Gal-I levels but upregulates BACE1 in primary HUVECs. (A) Primary HUVECs were treated with TNF-α (50 ng/ml) for 24 h. After FITC-labeled SNA staining, α-2, 6 sialylation expression was examined by confocal fluorescence microscopy. Mock, HUVECs without treatment. (B) Flow cytometry was then used to determine α-2, 6 sialylation expression. The data are the mean±SD of three independent assays. The MFI was quantified using BD FACS Software. NC, HUVECs unstained with FITC-labeled SNA. Mock, HUVECs without TNF-α treatment. (C) Western blotting was conducted to characterize ST6Gal-I and BACE1 levels in HUVECs after TNF-α treatment. The data are the mean±SD of three independent assays (**$p<0.01$). ns, not significant.

Fig.2A

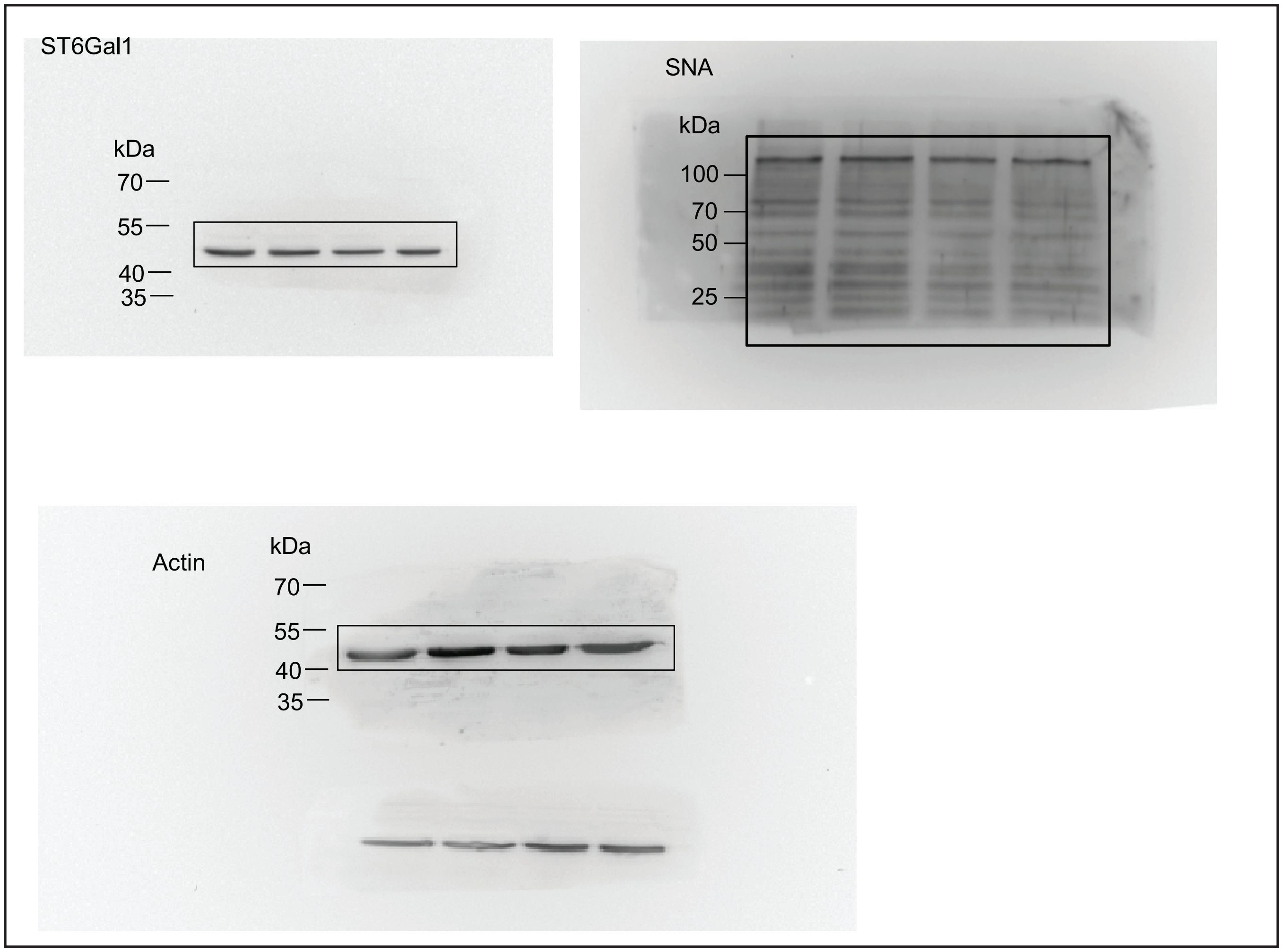

Fig.3A

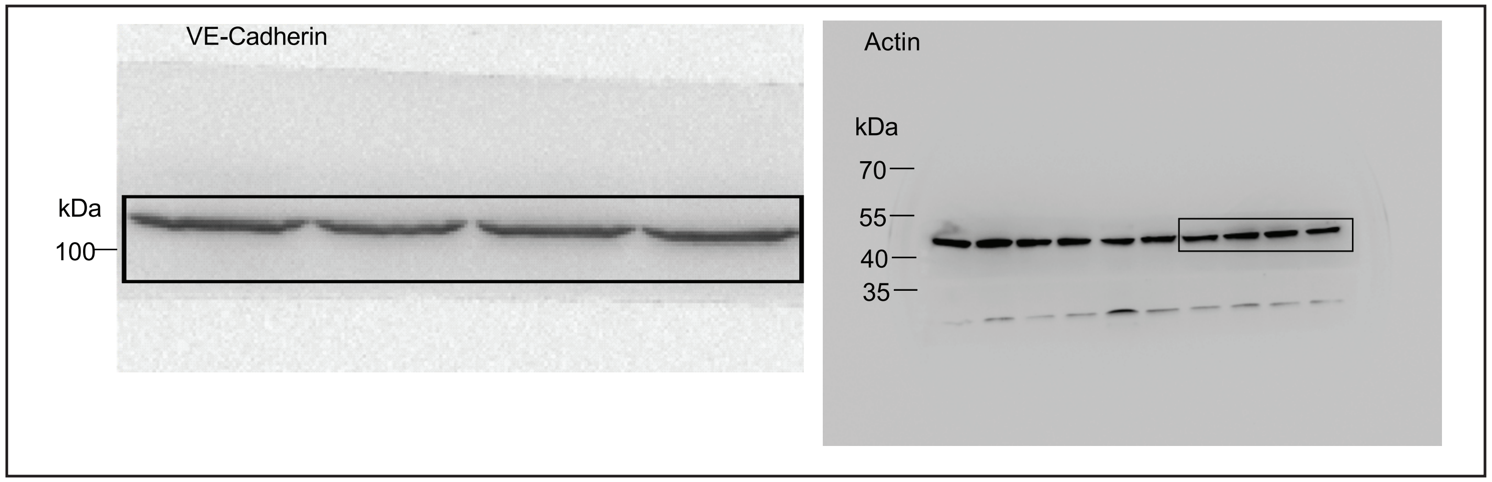

Fig.3B

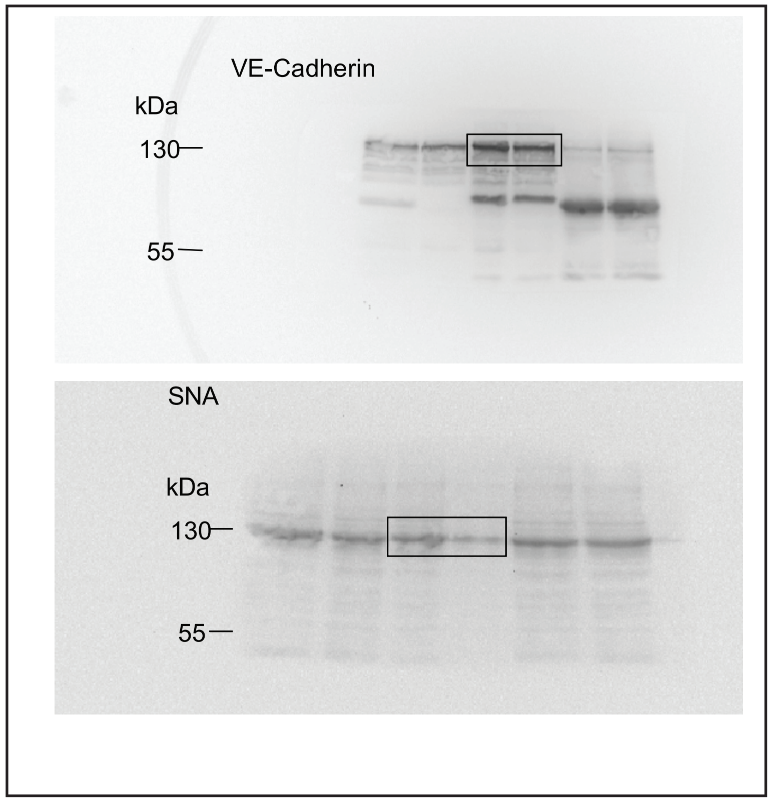

Fig.3C

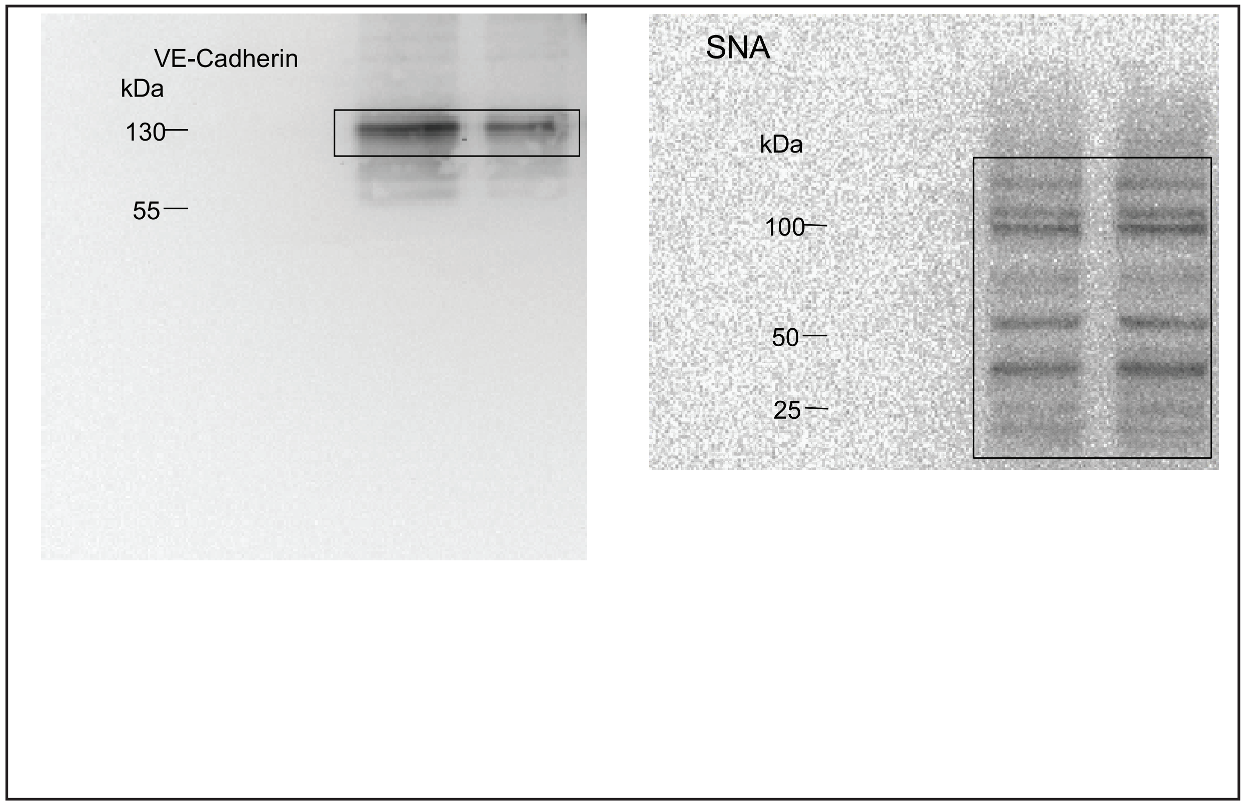

Fig.4A

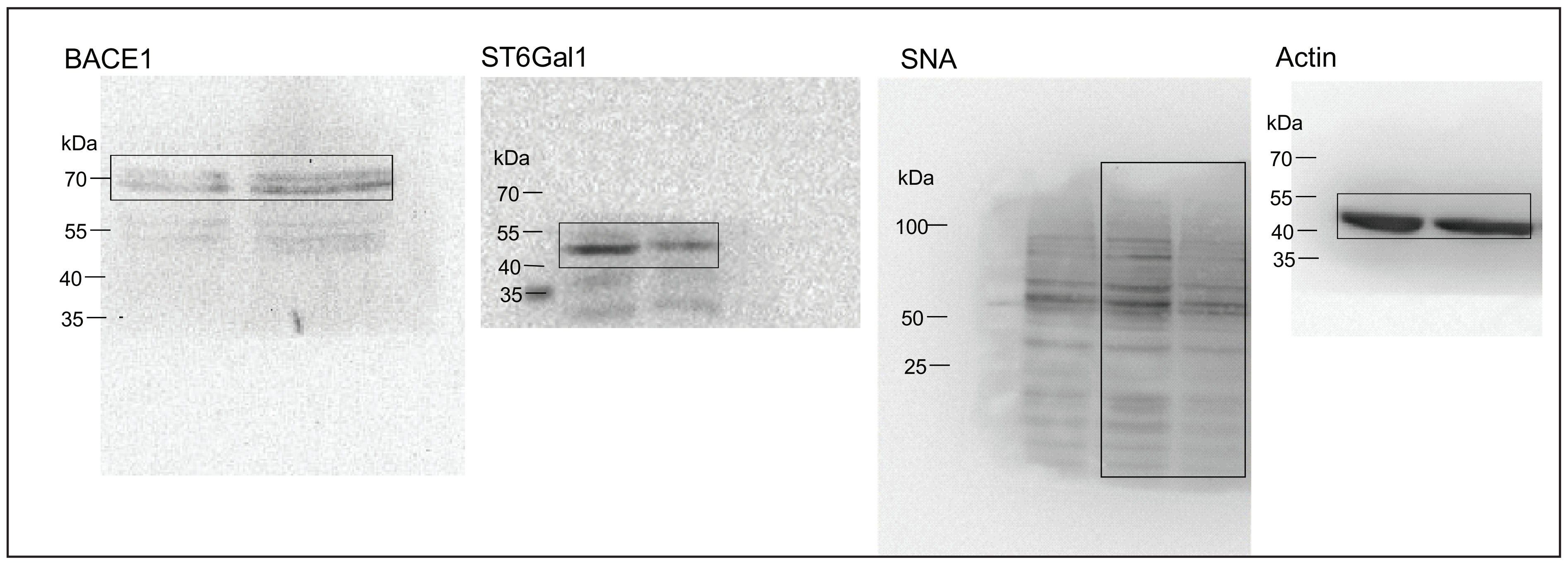

Fig.4B

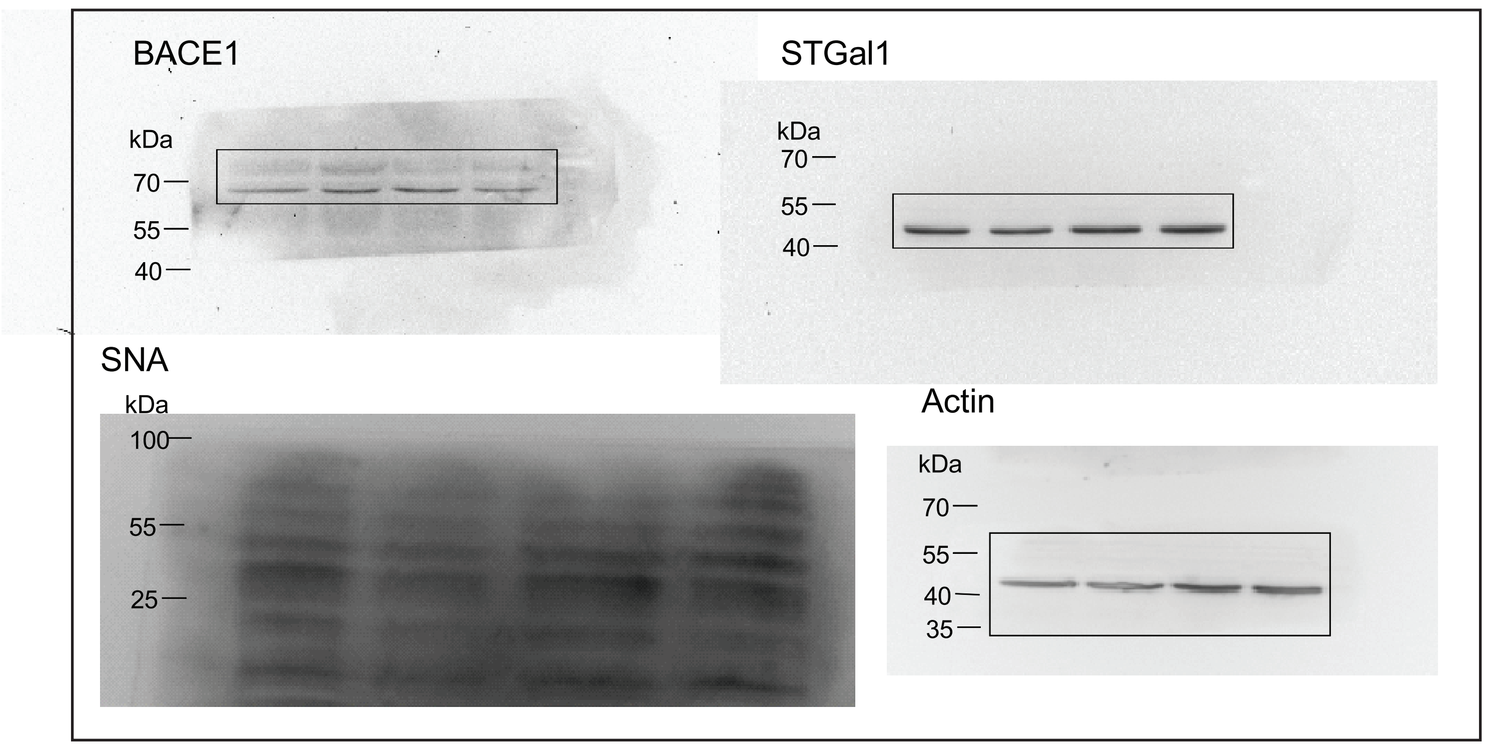

Fig.4D

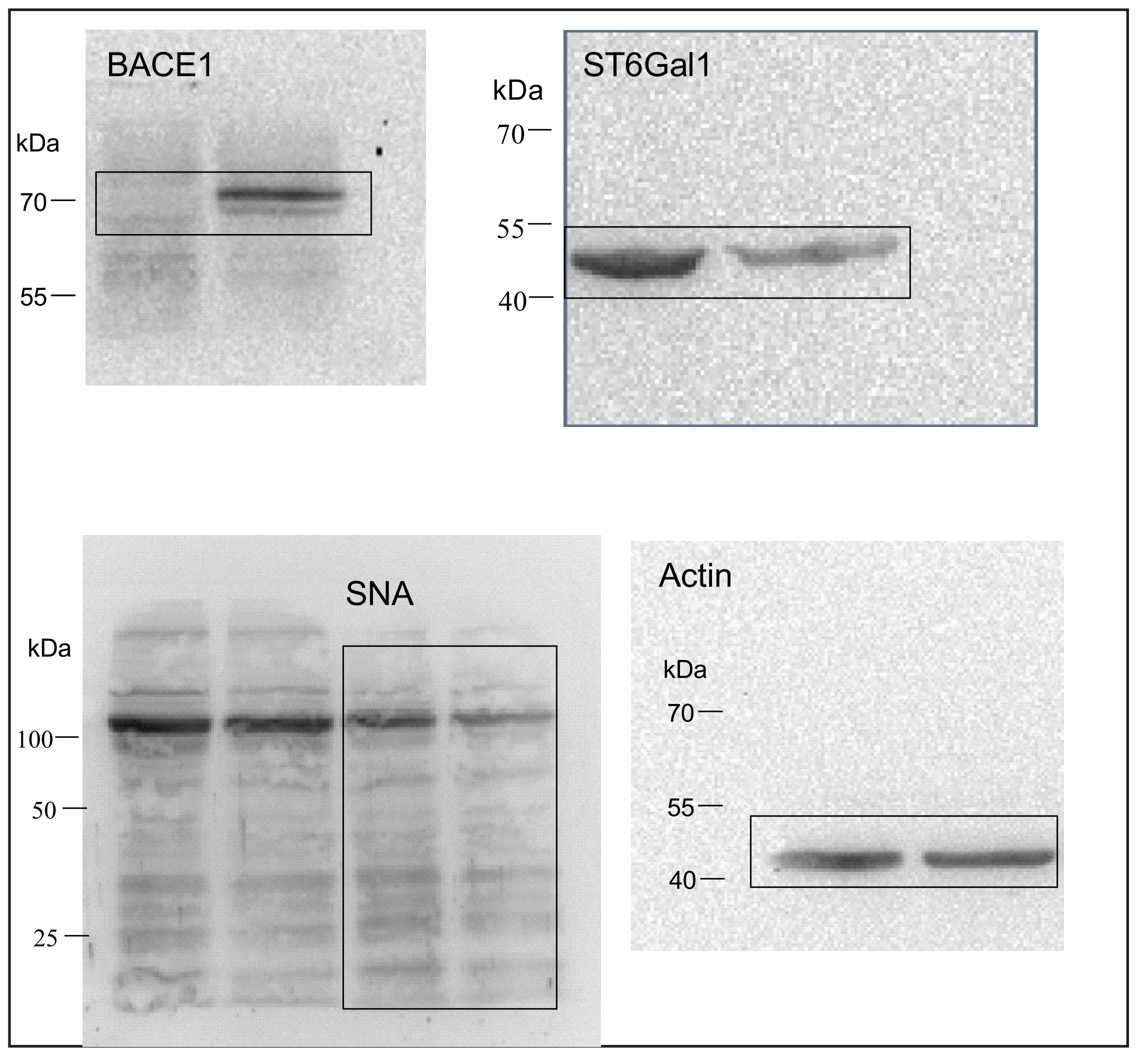

Fig.4F

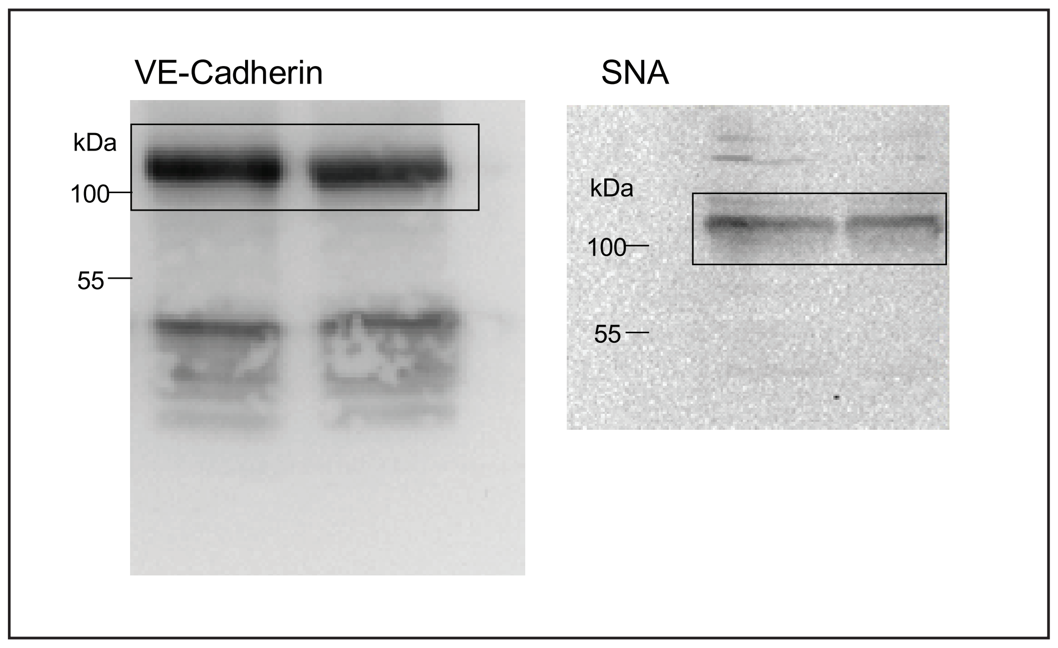

Fig.6A

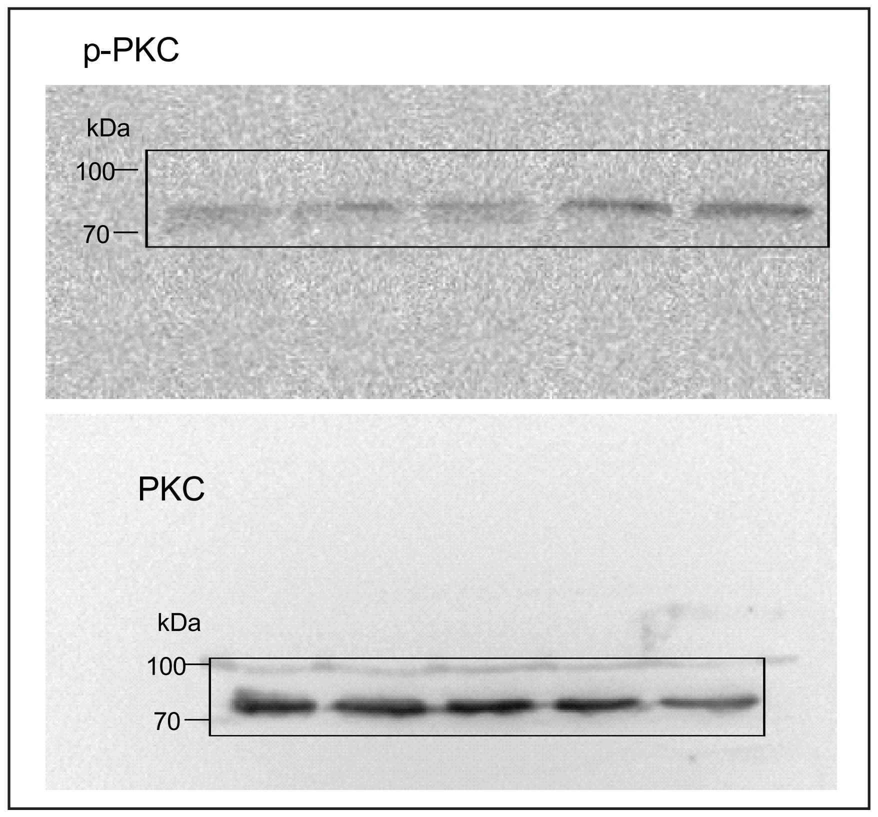

Fig.6B

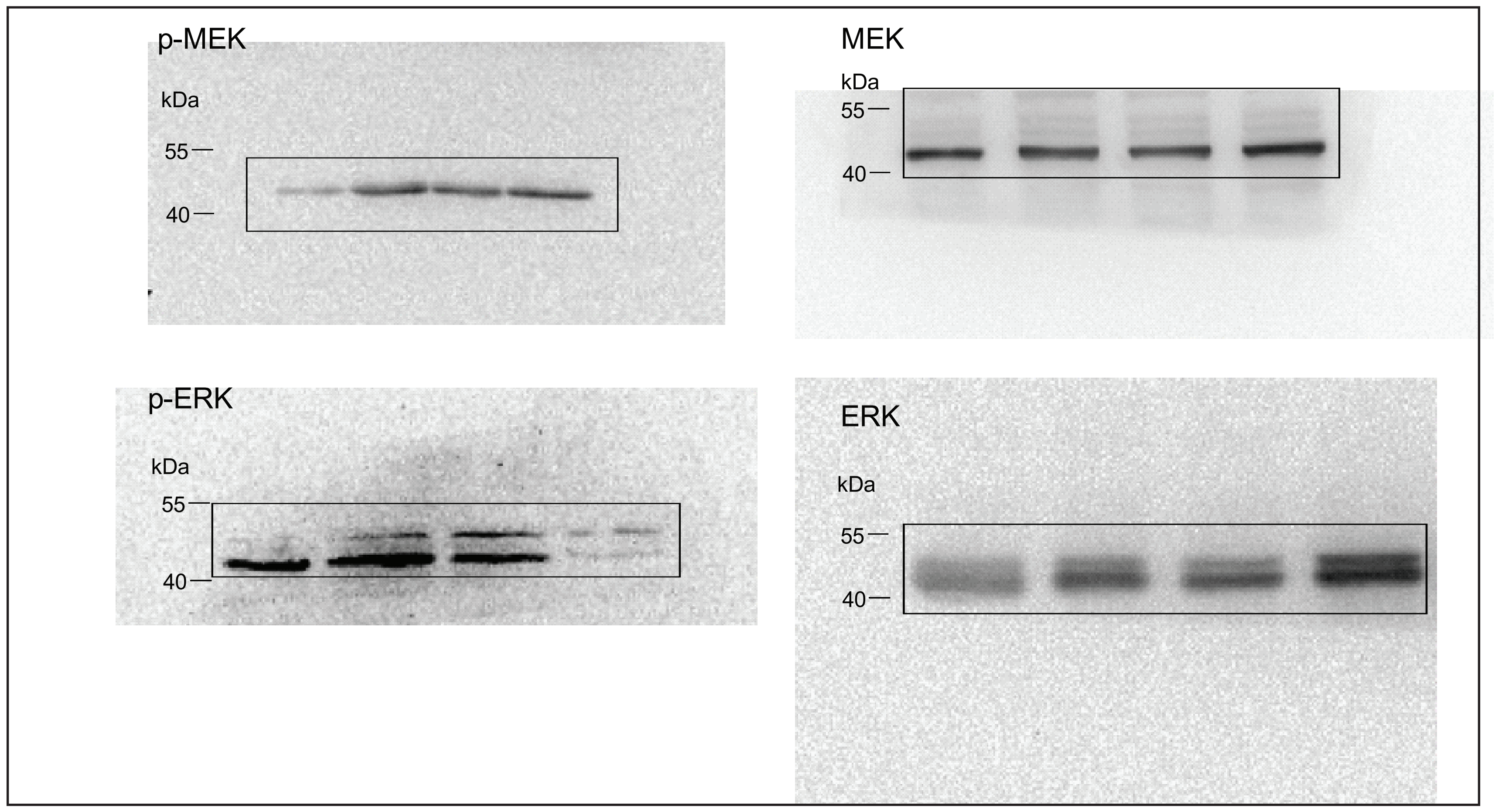

Fig.6C

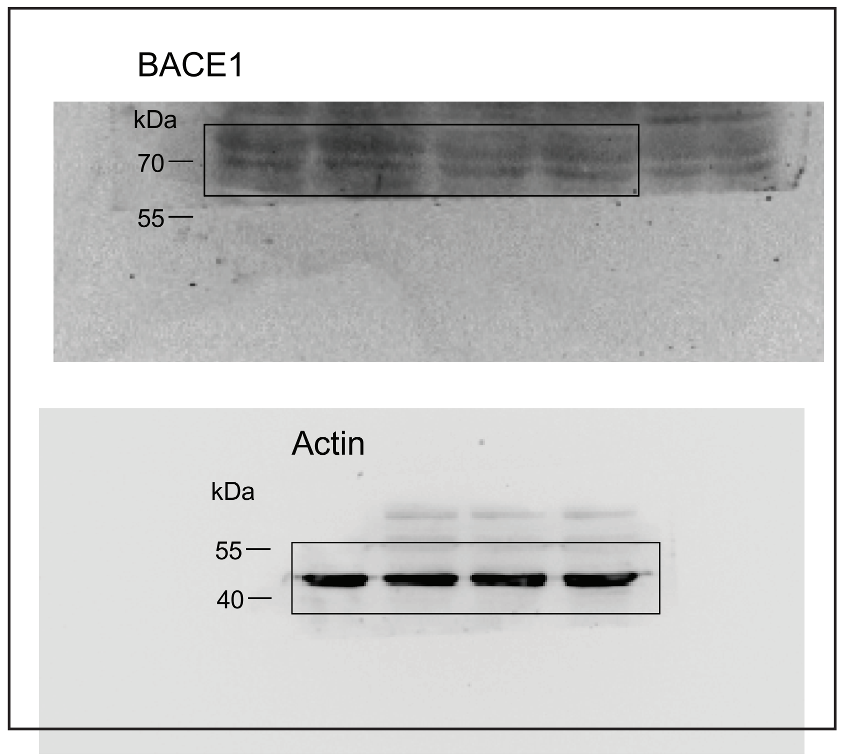

Fig.6D

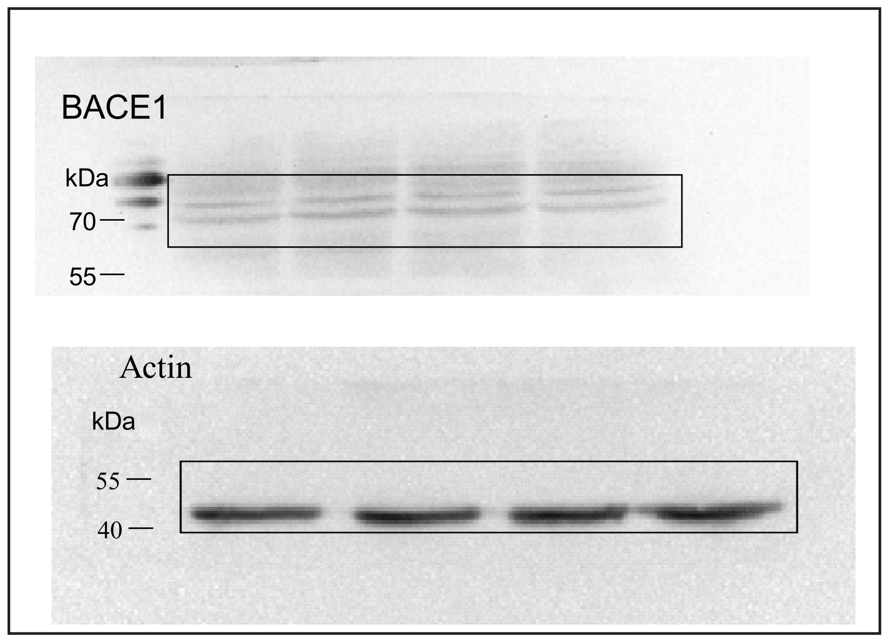

Supplement: Supplementary Information [file srep40256-s1.pdf]
